# Health facility readiness to care for high risk newborn babies for early childhood development in eastern Uganda

**DOI:** 10.1186/s12913-022-07693-0

**Published:** 2022-03-05

**Authors:** Gertrude Namazzi, Helena Hildenwall, Grace Ndeezi, Paul Mubiri, Christine Nalwadda, Angelina Kakooza-Mwesige, Peter Waiswa, James K. Tumwine

**Affiliations:** 1 Department of Health Policy, Planning and Management, School of Public Health, College of Health Sciences, Makerere University, Mulago Hill Road, P. O. Box 7072, Kampala, Uganda; 2 Department of Nursing, School of Health Sciences, College of Health Sciences, Makerere University, Kampala, Uganda; 3 Astrid Lindgren Children’s Hospital, Karolinska University Hospital, Stockholm, Sweden; 4 Health Systems & Policy, Department of Global Public Health, Karolinska Institutet, Stockholm, Sweden; 5 Department of Clinical Science, Intervention and Technology (CLINTEC), Karolinska Institutet, Stockholm, Sweden; 6 Department of Paediatrics and Child Health, School of Medicine, College of Health Sciences, Makerere University, Kampala, Uganda; 7 Department of Community Health and Behavioral Sciences, School of Public Health, College of Health Sciences, Makerere University, Kampala, Uganda; 8 School of Medicine, Kabale University, Kabale, Uganda

**Keywords:** Neurodevelopment, Facility readiness, High risk newborns, Essential newborn care, Birth asphyxia, Preterm

## Abstract

**Background:**

The neonatal mortality rate in Uganda has barely changed over the past decades, estimated at 28/1000 and 27/1000 live births in 2006 and 2016 respectively. The survivors have a higher risk of developing neurodevelopmental disabilities (NDD) due to brain insults from perinatal complications related to poor quality of health services during pregnancy, around the time of birth, and during the postnatal period. This study aimed to assess health facility readiness to care for high risk newborn babies in order to inform programming that fosters early childhood development in eastern Uganda.

**Methods:**

A cross sectional study of 6 hospitals and 10 higher level health centers that offer comprehensive maternal and newborn care was carried out in February 2020 in eastern Uganda. A World Health Organization Service Availability and Readiness Assessment tool (SARA) was adapted and used to assess the health facility readiness to manage maternal and neonatal conditions that are related to NDD. In addition, 201 mothers of high risk newborn babies were interviewed on their satisfaction with health services received. Readiness scores were derived from percentage average facilities with available infrastructure and essential medical commodities to manage neonatal complications. Descriptive statistics were computed for client satisfaction with service provision, and p values used to compare private not for profit to public health facilities.

**Results:**

There was limited availability in numbers and skilled human resource especially the neonatal nurses. Hospitals and health centers scored least in preterm and hypothermia care, with averages of 38% and 18% respectively. The highest scores were in essential newborn care, with readiness of 78% and 85% for hospitals and health centers, followed by resuscitation at 78% and 77%, respectively. There were no guidelines on positive interaction with newborn babies to foster neurodevelopment. The main cause of admission to neonatal care units was birth asphyxia followed by prematurity, indicative of intrapartum care challenges. The overall client satisfaction with health services was higher in private not for profit facilities at 91% compared to public hospitals at 73%, *p* = 0.017.

**Conclusion:**

Health facility readiness was inadequate in management of preterm complications. Efforts should, therefore, be geared to improving availability of inputs and quality of emergency obstetric and newborn care in order to manage high risk newborns and reduce the burden of NDD in this setting.

## Background

Globally, 2.5 million newborn babies die annually due to preventable causes [1]. Complications of birth including prematurity and low birth weight (LBW), Hypoxic Ischemic Encephalopathy/asphyxia, and neonatal sepsis account for 80% of all neonatal deaths. The survivors are at risk of neurodevelopmental disability (NDD) [2, 3]. NDDs are characterized by deficits in one or a combination of the following domains during the early period of a child’s growth: motor, and sensory functionality, attention, socio-emotional behavior, memory, and language [4]. The period from pregnancy to three years is the most critical phase for child brain development. Nurturing care that ensures early child development starts before birth and comprises of: health, nutrition, security and safety, responsive caregiving, and opportunities for early learning. The quality of health service provision and a supportive environment are pivotal to neurodevelopment during pregnancy, childbirth and postnatal period [5, 6].

Over 80% of the global birth complications occur in low income countries (LICs) with very limited access to neonatal intensive care units and well equipped special newborn care units (SNCU) [7]. Yet well-functioning newborn care units would save over 70% of newborn deaths and prevent disabilities due to prematurity and birth complications [8]. The majority of NDDs are related to insults due to poor quality of health services during pregnancy, around time of birth, and during the postnatal period [9]. Improvements in quality of maternal and newborn health services provided at health facility level, coupled with follow-up care is critical for early childhood development (ECD) and hence human capital productivity [5].

Small and sick newborn babies/high risk babies (HRB) require provision of timely quality services including feeding, warmth for the low birth weight (LBW) and preterms, phototherapy and safe oxygen support in case of hypoxemia [7]. Some of the recommended high impact and cost effective neonatal interventions for low resource settings include Kangaroo Mother Care (KMC) and Helping Babies Breath (HBB) [10–12]. KMC, if immediately initiated at the health facility and continued after discharge, was found to be beneficial in curbing NDD [13]. In addition, HRB should be protected from acquiring infections, and effective and adequate treatment with parenteral antibiotics where infection occurs. However, coverage of effective interventions is still limited [14], and health system bottlenecks limit the quality of services provided to the vulnerable newborn babies in LIC setting.

There is evidence that health facility contextual factors are influential in the level of quality of care (QoC) provided [16]. The Donabedian quality of care framework considers three components including the structure/inputs, processes, and health care outcomes [17]. Adequate knowledgeable and skilled health workers, availability of essential medicines and equipment, and an enabling/motivating environment are particularly important in saving the HRBs as well as preventing disabilities and enabling the vulnerable infants to thrive [5]. Major constraints in the workforce, financing, and service delivery for maternal and newborn care in LICs were reported by previous scholars [18–21]. For instance, in LICs the average nurse/midwife ratio per 10,000 population is only 7 compared to 57 in developed countries [7]. The limited numbers of, and skilled staff result in suboptimal maternal and newborn care which is unable to foster early child development [22, 23]. For example, a significant proportion of newborn babies in LICs have delayed breastfeeding, poor cord care, and are not assessed for potential danger signs before discharge following birth [24]. Quality of care gaps in resource constrained settings must therefore be addressed to realize reduction in neonatal deaths and ensure those that survive do thrive and attain their full developmental potential [25, 26].

In Uganda, while the policy environment is conducive for improved quality of care for the newborn, translation into practice is still a big challenge that requires better understanding of barriers in order to design contextually appropriate interventions [27] for not only newborn survival but for proper growth and development [28]. Although institutional deliveries in Uganda have increased from 42% in 2006 to 73% in 2016, this has not translated into reduction of neonatal mortality estimated to be 28/1000 in 2006 and 27/1000 live births in 2016 [29, 30]. Recent studies also indicate that there is a high prevalence of NDD in the country, and most of the associated factors including asphyxia, post-neonatal complications, and malnutrition among preterm infants are preventable [13, 31]. There is currently an increased scale up of neonatal care units in the country but the quality of care provided for management of HRBs to prevent mortality and NDD is not clear. This study therefore, aimed at assessing health facility readiness to care for high risk newborn babies in order to inform programming that fosters early childhood development in eastern Uganda.

## Methods

### Study Design and Setting

A cross sectional study was carried out in February 2020 in six hospitals and ten higher level health centers (HC IVs) in Busoga region in eastern Uganda. This was before the first Covid-19 case had been identified in the country. In Uganda Health Centres (HC) vary in their care provision across three levels – HC II, HC III, and HC IV. The higher level HC IVs offer comprehensive maternal and newborn care for HRBs. Busoga region has a population of about four million people, with a NMR estimated at 30/1000 live births in 2015[32]. The region is served by eleven hospitals [33].

The hospitals included in this study were 4 public and 2 ‘private not for profit’ health facilities (PNFP). All the HCIVs were government owned health facilities. One of the public hospitals was a regional referral hospital to which the rest of the general hospitals refer complicated cases. All the six hospitals were sites for the Preterm Birth Initiative (PTBi) study which was conducted between 2016 and 2019. The PTBi study aimed at reducing preterm morbidity and mortality through four intervention components: Data strengthening, use of the modified WHO Safe Childbirth Checklist, health provider training and mentorship, and use of collaborative quality improvement approach [34]. The PTBi study also provided some equipment and supplies at the start of implementation to address the critical gaps identified during the baseline study.

In the current study, the facilities were assessed to determine their readiness for care of HRBs: availability of inputs based on standards and clients’ experiences of the maternal and newborn care received. The health facilities were selected on the basis of being high volume facilities that are mandated to offer emergency obstetric and newborn care. We selected public and PNFP hospitals, and health centers IVs. This allowed us to assess the facility readiness based on the variation of the type of facilities in the region.

### Data collection

The WHO Services Availability and Readiness Assessment (SARA) tool, previously used by the PTBi study, was adapted based on the matrix developed by Moxon et al. 2018 [35], and used to assess the readiness of included facilities to care for HRBs. The adaptation of the SARA tool involved adding infrastructure for thermoregulation beyond KMC, and management of jaundice, use of a continuous positive airway pressure (CPAP) ventilation, and neurodevelopmental support. The inputs for neurodevelopmental support included: availability of cyclic lights, sound control measures, and guidelines for positive interactions with newborns and communication with carers. The infrastructure assessed included space for special care and resuscitation, stabilization and KMC. The staffing levels and availability of the skilled cadres were determined. An inventory was taken of equipment and commodities including nasal gastric tubes for feeding, availability of antibiotics for treatment of neonatal bacterial infection, intravenous fluids, oxygen, use of pulse oximetry, and use of a phototherapy machine for effective case management of pathological jaundice. The inventory data were collected by the first author, with the support of one research assistant, using a paper checklist written in English.

The experience of service provision for HRBs from the client perspective was assessed through client exit interviews in the hospitals. The HRBs were defined as: babies with APGAR score of less than 7 five minutes after birth, preterms with a gestation age of less than 37 weeks at birth, babies with a birth weight of less than 2500 g at birth, and infections characterized by either convulsions, failure or cessation of feeding, fast breathing of > 60 breaths per minute, severe chest in-drawing, temperature > 37.5 °C or < 35.5 °C, movement only when stimulated or no movement at all. Additionally, HRBs included those with pathological jaundice: a condition where a term newborn baby presents with jaundice within 24 h after birth, or the total serum bilirubin level is higher than 17 mg/dl in infants 25 to 48 h old, and the infant has signs and symptoms of serious illness. On average, 250 HRBs were admitted in the SNCU on a monthly basis from the six hospitals [36]. After excluding 10 runaway cases, 14 patients referred to other facilities and 25 deaths, 201 mothers with HRBs were included in the study. Proportionate to size sampling methods were used to distribute the sample size across the six hospitals.

Six research assistants with medical background who were trained for two days and supervised by the first author, were attached to the hospitals (one per hospital) for a month. The research assistants liaised with the nurses working in the maternity and special newborn care units of the respective hospital and were informed of the potential discharges to enable interviews to be conducted immediately after discharge. On discharge, mothers with HRBs were interviewed on the care their babies received and how it was provided using the exit interview tool in *Lusoga*, the local language. Satisfaction with the services mothers received was captured by questions regarding the attitude of health providers, consultation time given to them, waiting time, general cleanness of the premises and on specific care during the delivery and postnatal period. In addition, mothers were asked whether they were given information on how to care for their babies after discharge, any counselling on danger signs and feeding, and details on when to come back for review of the babies. We ensured that participants did not wait for more than 10 min before interviews following discharge from the SNCU.

### Data management and analysis

Facility readiness to manage maternal and neonatal conditions that result in NDD was determined by the availability of infrastructure, medical commodities, skilled providers and client satisfaction with service provision. These were based on the list of evidence-based treatments (inclusive of items for diagnosis, treatment, and monitoring) for the common neonatal conditions developed by Morgan and team (essential routine newborn care; neonatal resuscitation; feeding and hypothermia; respiratory distress/apnea of prematurity; infection, convulsions and jaundice) [20], and on the WHO quality of care standards on developmental support for sick and small newborn babies [6]. Neonatal complications are known to be the main causes of neonatal deaths and also responsible for neonatal developmental disabilities among survivors. In addition, availability of resources for antenatal and emergency obstetric care (EmOC) were included given their critical role in neonatal survival and developmental potential during prenatal and intrapartum period.

Data from the SARA tool and exit interviews were entered in ACCESS with consistence checks and later exported to STATA version 15 for analysis. Descriptive statistics using frequencies, percentages, means, and standard deviations were used to summarize the data stratified by type of facility. Readiness scores were derived from proportions of facilities with availability of essential equipment, supplies and medicines for care of HRBs. The scores were then compared across the public hospitals, PNFP, and HCIV facilities.

The satisfaction of mothers of HRBs was assessed on several services received. We considered satisfaction to include those who mentioned ‘very satisfied’ and ‘satisfied’ for each variable. Chi square test statistic was computed to determine whether there was any difference in satisfaction between the Public and PNFP hospitals.

### Ethical considerations

Ethical approval to conduct the study was obtained from the Higher Degrees and Research Ethical Committee (HDREC) of Makerere University School of Health Sciences (Ref. 2017- 011) and Uganda National Council of Science and Technology (#SS4600). Permission to access the health facilities was obtained from the district health authorities and the hospital administration prior to facility assessment and exit interviews. Written informed consent was obtained from all mothers of HRBs before data collection.

## Results

### Health facility characteristics

All the six hospitals were conducting deliveries 24 h a day, with capacity to do a caesarian section and with a neonatal special care unit including a dedicated unit for KMC. Although all the HCIVs were conducting deliveries 24 h a day only six were able to carry out Caesarian sections, two were unable due to inadequacies of theatre, while another two lacked anesthetists and relied on medical officers for giving anesthesia. All the health facilities had basic amenities such as electricity and running water. The average monthly deliveries were more at the public hospitals (450) than at the PNFP (79) and HCIVs (106) (Table 1). All the health facilities had medical officers attached to maternity and SNCU units, but only the regional referral hospital and the high volume district level hospital had obstetricians and pediatricians. The clinical officers at hospital level were only attached to the general outpatient department and did not work in the antenatal or maternity units, while at HCIV they could work in other inpatient departments rather than maternity. There was only one neonatal nurse based at the regional referral hospital. The rest of the health facilities had midwives that run the NSCU, and these were also the same midwives that conducted deliveries in the general hospitals and HCIVs. The average monthly number of HRBs admitted in the public hospitals was 61 compared to 15 in the PNFP, and 12 in HCIVs. There were no guidelines in any facility for positive interactions with the HRBs and for communication with the caregivers on how to ensure proper neurodevelopment of the vulnerable newborn babies.

**Table 1:** Health facility characteristics: utilization, infrastructure and human resource

Characteristics	Items/requirements	Public Hospitals *n* = 4	PNFPs Hospitals *n* = 2	All Hospitals *n* = 6 (%)	HCIVs (Public) *n* = 10 (%)
**Infrastructure**					
	Dedicated KMC unit with amenities for rooming in	4	2	6 (100)	4 (40)
	Running water	4	2	6 (100)	8 (80)
	Soap/disinfectant	4	2	6 (100)	10 (100)
	Electricity	4	2	6 (100)	10 (100)
**Average monthly health facility utilization**					
	ANC	1,179	388	915	431
	Delivery	450	79	326	106
	HRB (Small & Sick newborn) admissions to the NSCU	61	15	45.7	12
**Staffing/Human Resource**	**Attached to antenatal, maternity, Postnatal, NSCU and peadiatric ward**				
Numbers	Obstetricians	3	0	3	
	Peadiatricians	3	0	3	
Average number	Medical Officers	5	3.5	4.6	2
	Anesthetists	2.8	1.5	2.3	0.4
	Midwives	12.3	10	11.5	6.1
	Nurses/midwives attached to NSCU	2.8	1.4	2.3	00
	NSCU Nurse to newborn ratio	1:21	1:11	1:20	00
**Developmental supportive care**					
	Guidelines on Positive interactions with newborns and communication with carers	00	00	00	00

### Goal oriented antenatal care and delivery care

Readiness for provision of ANC was high in HCIVs (83%) but generally low in hospitals with an average score of 58% (Table 2). The readiness was lower in public health facilities due to lack of access to ultrasonography at the facilities during pregnancy, lack of examination/flash light, and limited availability of diagnostic equipment particularly for hemoglobin and blood sugar level estimation. Two (50%) of the public health facilities did not have a single functioning blood pressure machine in the antenatal departments, only one out of the four public hospitals had stock of ferrous sulphate tablets, and one had run out of stock of folic acid tablets.

**Table 2:** Antenatal care and EmOC guidelines, supplies and medicines

Stage of care	Items/requirements	Public hospitals *n* = 4	PNFPs Hospitals *n* = 2	All hospitals *n* = 6 (%)	HCIVs n = 10 n (%)
**Antenatal care**					
	Guidelines	3	2	5 (83.3)	7 (70)
	Visual aids for counselling	4	2	6 (100)	9 (90)
	Adult weighing scale	4	2	6 (100)	10 (100)
	Blood Pressure machine	2	2	4 (67)	9 (90)
	Ferrous sulphate	1	2	3 (50)	10 (100)
	Folic acid	3	2	5 (83.3)	10 (100)
	Oral Antibiotics (Amoxycillin, etc.)	4	2	6 (100)	9 (100)
	Intermittent Preventive Therapy (Fansidar^a^)	3	2	5 (83.3)	10 (100)
**Antenatal care**	**Diagnostics (for syphilis, malaria, blood sugar, Urine tests)**				
	Syphilis testing	3	2	5 (83.3)	10 (100)
	Malaria testing	4	2	6 (100)	10 (100)
	Urine protein test	2	0	2 (33.3)	8 (80)
	Urine Glucose test	2	0	2 (33.3)	9 (90)
	Blood glucose test	1	0	1 (16.7)	8 (80)
	Any rapid test for hemoglobin	1	0	1 (16.7)	9 (90)
	Ultrasonography	0	2	2 (33.3)	2 (20)
	Examination light (flashlight)	0	0	0 (00)	5 (50)
	Fetal doppler	1	1	2 (33.3)	7 (70)
	Thermometer	1	2	3 (50.0)	8 (80)
	Visual privacy only	1	1	2 (33.3)	8 (80)
**Overall mean score**	**ANC**	**2.1 (52.5)**	**1.4 (68.4)**	**3.4 (58.3)**	**8.3 (83)**
**EmOC**					
	Guidelines for emergencies (Pre/Eclampsia, Postpartum hemorrhage, etc.)	3	2	5 (83.3)	9 (90)
	Partographs for monitoring labor	4	2	6 (100)	10 (100)
	Oxytocin to augment labor	3	2	5 (83.3)	9 (90)
	**Items available and functioning in the delivery area**				
	Ultrasound machine	1	0	1 (16.7)	4 (40)
	Blood pressure cuff	4	2	5 (83.3)	9 (90)
	Resuscitation table with heat source	2	1	3 (50.0)	3 (30)
	Pulse oximeter with neonatal probe	2	0	1 (16.7)	5 (50)
	Filled oxygen canisters	1	0	1 (16.7)	5 (50)
	Infant weighing scale	2	1	3 (50.0)	9 (90)
	Suction bulb	4	2	6 (100)	9 (90)
	Soap or hand disinfectant	3	2	5 (83.3)	10 (100)
	IV/IM Antibiotics	4	2	6 (100)	8 (80)
	IM/IV Magnesium Sulphate (anticonvulsant)	3	2	5 (83.3)	8 (80)
	Antihypertensive e.g. hydralazine	4	2	6 (100)	5 (50)
	Vacuum extractor-for assisted delivery	2	1	3 (50)	5(50)
	Ability to do surgery (C/S): MO/OBGY, Theatre, Anesthetics, Surgical instruments, etc	4	2	6 (100)	8 (80)
	Blood transfusion	4	2	6 (100)	8 (80)
	Referral protocols	2	0	2 (33.3)	9 (90)
	Resuscitation algorithm	2	2	4 (66.7)	7 (70)
**Overall mean score**	**EmOC + Blood transfusion**	**2.8 (71.1)**	**1.4 (71.1)**	**4.2 (70.0)**	**7.4 (74)**

Fansidar^a^ = sulphadoxine/pyrimethamine, *EmOC* Emergency Obstetric Care

The readiness average scores for EmOC and blood transfusion services were 70% in hospitals and 74% in HCIVs (Table 2). However, there were limitations in availability of oxygen, found in only 2 public hospitals and pulse oximeters were available in only one public hospital. Similarly, there was lack of access to ultrasound scan, which was only available in one general high volume hospital and four of HCIVs. Although resuscitation tables for newborn babies existed in all the health facilities, they lacked overhead heaters. The resuscitation algorithm and referral protocols were available in most (70%) of the HCIVs but in only two (50%) of the public hospitals while both PNFP hospitals did not have referral protocols.

### Essential routine newborn care and resuscitation

Readiness scores for essential routine newborn care were higher in HCIVs (85%) compared to hospitals at 78% (Table 3). However, the readiness for resuscitation was not so different in the hospitals (78%) and in HCIVs (77%). Only two hospitals (one PNFP and one public) had umbilical cord ties. Most health facilities were improvising by using the end parts of gloves as cord ties. The resuscitation area lacked a heat source in half of hospitals and HCIVs. Similarly, only half of the hospitals had the pulse oximeters with accompanying probes.

**Table 3:** Postnatal care including resuscitation, feeding and infection control, supplies and medicines

Immediate PNC	Items/requirements	Public hospitals *n* = 4	PNFPs hospitals *n* = 2	All hospitals *n* = 6 (%)	HC IVs *n* = 10 n (%)
**Essential NB care**					
	Clean blades	4	2	6 (100)	10 (100)
	Cord ties/ligatures	1	1	2 (33.3)	5 (50)
	Tetracycline ointment	4	2	6 (100)	10 (100)
	Prevention of mother to child transmission of HIV	4	2	6 (100)	10 (100)
	Weighing scale	4	2	6 (100)	9 (90)
	Referral guidelines	2	0	2 (33.3)	7 (70)
**Overall mean score**	**Essential newborn care**	**3.2 (80.0)**	**1.5 (75.0)**	**4.7 (78.3)**	**8.5 (85)**
**Resuscitation**					
	Neonatal resuscitation algorithm	2	2	4 (66.7)	7 (70)
	Resuscitation area with heat lamp/source	2	1	3 (50.0)	5 (50)
	Ventilation Bag	4	2	6 (100)	9 (90)
	Mask (Term & preterm)	4	2	6 (100)	9 (90)
	Suction device	4	2	6 (100)	10 (100)
	Pulse oximeter with a probe	2	1	3 (50.0)	6 (60)
**Overall mean score**	**Resuscitation**	**3 (75.0)**	**1.7 (85.0)**	**4.7 (78.3)**	**7.7 (77)**
**Infection control and management + Convulsions**					
	Water & soap/hand sanitizer	4	2	6 (100)	10 (100)
	7.1% Chlorhexidine for cord care	0	0	0 (00)	00
	C-Reactive protein testing	1	0	1 (16.7)	00
	Stethoscope	4	2	6 (100)	8 (80)
	Disposable gloves	4	2	6 (100)	6 (60)
	Lancets (neonatal size)	0	0	0 (00)	00
	Thermometer	3	2	5 (83.3)	9 (90)
	Multi-function monitors	2	0	2 (33.3)	00
	IV Cannulas	4	2	6 (100)	8 (80)
	IV giving sets/tubing	4	2	6 (100)	7 (70)
	Newborn weighing scale	4	2	6 (100)	10 (100)
	Inj. Gentamycin	0	2	2 (33.3)	8 (80)
	Inj. Ampicillin/penicillin	2	2	4 (66.7)	10 (100)
	Glucometer	4	0	4 (66.7)	5 (50)
	Glucose test strips	1	0	1(16.7)	5 (50)
	Anticonvulsant-Phenobarbitone	4	2	6 (100)	7 (70)
	Infusion pump	0	0	0 (00)	00
	Burettes	2	2	4 (66.7)	00
	Dextrose 10%	2	2	4 (66.7)	00
	Guidelines for referral	2	0	2 (33.3)	5 (50)
**Overall**	**Overall infection control, management + Convulsions**	**2.4 (60.0)**	**1.2 (60.0)**	**3.6 (60.0)**	**4.9 (49)**
**Feeding**					
	Water & soap/sanitizer	4	2	6 (100)	10 (100)
	NB weighing scale	4	2	6 (100)	10 (100)
	Tape measure in NSCU	3	2	5 (83.3)	6 (60)
	IV cannulas	4	2	6 (100)	10 (100)
	Burettes	2	2	4 (66.7)	0 (00)
	Dextrose-10%	2	2	4 (66.7)	0 (00)
	Ringers Lactate	4	2	6 (100)	9 (90)
	Nasal gastric tube- neonatal size	4	2	6 (100)	4 (40)
	Syringes/cups for feeding	4	2	6 (100)	4 (40)
	Lancets	0	0	0 (00)	00
	Glucometer	4	0	4 (66.7)	5 (50)
	Glucose test strips	1	0	1 (16.7)	5 (50)
	Small & Sick newborn feeding guidelines	4	2	6 (100)	5 (50)
	Breast pumps	0	0	00	00
	Referral guidelines	0	0	00	00
**Overall**	**Feeding**	**2.7 (67.5)**	**1.3 (65.0)**	**4 (66.7)**	**4.9 (49)**
**Preterm & Hypothermia care**					
	Incubator or radiant warmer	4	2	6 (100)	6 (60)
	KMC bed/chair	1	0	1 (16.7)	00
	IV Aminophyline	4	2	6 (100)	6 (60)
	CPAP	1	1	2 (33.3)	2 (20)
	Phototherapy machine	2	1	3 (50.0)	4 (40)
	Surfactant	0	0	00	00
	Ear muffs/Earplugs for sound control	0	0	00	00
	Cyclic lights or light reducing goggles	0	0	00	00
**Overall**	**Overall Preterm & Hypothermia care**	**1.5 (37.5)**	**0.8 (40.0)**	**2.3 (38.3)**	**1.8 (18)**

### Infection control, management and convulsions readiness

Overall, the average score for infection control, management + convulsions readiness was 60% in hospitals, public and PNFP facilities, and about 50% in HCIVs (Table 3). None of the health facilities assessed had chlorhexidine for cord care to prevent infection, and none of the facilities had C-reactive protein tests for assessing the possibility of infection. Similarly, all the facilities had no neonatal lancets for taking off blood for laboratory testing. They were using ordinary needles to prick the neonates. Gentamycin, an essential first line antibiotic for treatment of neonatal septicemia, was out of stock in all public hospitals. Only two high volume public hospitals had the multi-functional monitors to monitor the vital functions of sick newborn babies. None of the health facilities had an infusion pump, and only two public facilities had burettes for controlled provision of intravenous fluids, and dextrose (Table 3). None of the HCIVs had readily available 10% dextrose for treatment of hypoglycemia, and also lacked 50% dextrose to enable preparation locally.

### Hypothermia and preterm care

Readiness of all health facilities was poorest regarding preterm and hypothermia care. The overall average score was 38% for hospitals and 18% for HCIVs (Table 3). The poor score was due to limited facilities for sound and light control, lack of surfactant, and KMC beds or chairs. Only the referral hospital had one KMC bed provided by the KMC study. The rest of the health facilities were using ordinary beds and plastic chairs for mothers to practice KMC. In addition, only about half (3 hospitals and 4 HCIVs) of the health facilities had phototherapy machines for care of jaundiced newborn babies.

### Feeding of sick and small newborn babies

The overall score for feeding of sick and small babies was about 50% in HCIVs and 67% in hospitals, and this was similar between public (67%) and PNFP (65%) hospitals (Table 3). The lancets, glucose test strips, breast pumps and referral guidelines were lacking in almost all health facilities.

### Characteristics of participants for the exit interview

A total of 201 mothers (166 from public and 35 from PNFP health facilities) were interviewed during exit interviews. There were significantly younger mothers (mean age 21.4 years SD ± 4.4) whose HRBs were admitted to the PNFP hospitals, compared to those in public facilities, *p* = 0.008) (Table 4). However, there was no difference in the period babies spent in the SNCU, with an average duration of about 5.3 days SD ± 4.3 in public hospitals compared to 4.9 days SD ± 2.3 in PNFP hospitals, *p* = 0.552. Similarly, there was no difference in the diagnosis captured on the discharge forms. Most babies had a diagnosis of asphyxia (37% in public facilities and 43% in PNFP hospitals) followed by preterm/LBW (28% and 26% respectively, *p* = 0.971).

**Table 4:** Description of participants for exit interviews

**Participant characteristics**	**Public HFs**	**PNFPs**	**p-value**
	*N* = 166	*N* = 35	*N* = 201
Average age of mother (SD)	24.1 (5.4)	21.4 (4.4)	0.008**
Age of baby in days (SD)	5.3 (4.3)	4.9 (2.3)	0.552
Male Sex	57.1	41.2	0.092
Average birth weight (SD)	2.6 (0.8)	2.2 (0.6)	0.006**
**Diagnosis on discharge form**			0.971
Asphyxia (difficulty in breathing)	62 (37.3)	15 (42.9)	
Preterm/LBW	46 (27.7)	9 (25.7)	
Neonatal infection	32 (19.3)	6 (17.1)	
Convulsions	3 (1.8)	0 (0.0)	
Jaundice	10 (6.0)	3 (8.6)	
Others	13 (7.8)	2 (5.7)	

### Client experience and satisfaction with service provision for high risk newborn babies

Overall, client satisfaction with the services provided, and satisfaction with waiting time, health facility cleanness, and providers’ attitudes were over 90% in PNFP hospitals, significantly higher compared to public hospitals (*p* < 0.05) (Table 5). However, satisfaction with privacy was not significantly different in the two settings (*p* = 0.303).

**Table 5:** Client satisfaction with health care services, knowledge of neonatal danger signs, counselling on newborn illness, and follow up care plans

**Variables**	**Public HFs** ***N* = 166 (%)**	**PNFPs** ***N* = 35 (%)**	**p-value**
**Client satisfaction**			
Overall satisfaction	121 (72.9)	32 (91.4)	0.017
Mean Waiting time (minutes)	42.5 (85.8)	16.9 (9.4)	0.075
Satisfaction with waiting time	109 (65.7)	34 (97.1)	< 0.001
Cleanness	117 (70.5)	35 (100.0)	< 0.001
Privacy	117 (70.5)	28 (80.0)	0.303
Time given	133 (80.1)	35 (100.0)	0.002
Respect by providers	117 (70.5)	33 (94.3)	0.002
**Counselling**			
One on one about condition of baby	142 (85.5)	35 (100.0)	0.010
Prognosis/possible complications	122 (73.5)	35 (100.0)	< 0.001
Danger signs	109 (65.8)	35 (100.0)	< 0.001
Nutrition	130 (78.3)	35 (100.0)	0.001
Breast feeding	153 (92.2)	35 (100.0)	0.130
Care at home	139 (83.6)	35 (100.0)	0.005
When to come back for review	152 (91.6)	35 (100.0)	0.136
Family planning	97 (58.4)	21 (60.0)	1.000
Husband involved during counselling	85 (51.2)	31 (88.6)	< 0.001
**Knowledge of newborn danger signs**			
Able to mention two danger signs	83 (50.0)	10 (28.6)	0.021
**Baby examined after delivery**			
Examined	162 (97.6)	35 (100.0)	1.000
After how long baby is examined			0.001
< 1 h	118 (71.1)	35 (100.0)	
1–6 h	30 (18.1)	00 (0.0)	
> 6 h	18 (10.8)	00 (0.0)	
**Frequency of examining baby**			0.884
Once a day	10 (6.1)	01 (2.8)	
Twice a day	151 (90.9)	34 (97.1)	
Sometimes/not daily	5 (3.0)	00 (0.0)	
**Immunization**			
Baby immunized before discharge	134 (81.0)	26 (74.3)	0.352
**Money paid, supplies/medicines bought**			
Bought medicines/supplies outside HF	87 (52.7)	10 (29.4)	0.013
Paid money to provider	46 (27.9)	10 (29.4)	0.856
Median amount paid (UG shillings)	20,000	100,000	

The results further revealed that counselling on the prognosis of baby’s condition, and on newborn danger signs was done for all mothers in PNFP hospitals compared to 74% and 65.8% respectively in public facilities. However, only 29% of mothers in PNFP facilities were able to mention at least two newborn danger signs compared to 50% of those in public facilities. The proportion of mothers counseled on family planning before discharge (60%) was similar in both settings.

All babies were reportedly examined within one hour after birth in PNFP hospitals compared to only 71% in public health facilities. In fact, almost all babies were examined at least twice per day in both settings. However, there were missed opportunities for immunization of the babies before discharge in both public (19%) and PNFP (26%) settings.

## Discussion

In this study we found that overall health facility readiness to care for HRBs was insufficient with the lowest preparedness within the area of preterm care and hypothermia where the scores were 38% in hospitals and 18% in HCIVs. Health facilities also scored low in readiness for the control and management of infections and convulsions. The readiness was highest within the fields of essential routine newborn care and neonatal resuscitation followed by feeding. The results are not surprising given the Ugandan Ministry of Health’s efforts focusing on essential routine newborn care and resuscitation through the HBB-plus intervention program throughout the country. Previous studies in the country also reported facility readiness to have been consistently highest for essential newborn care [20, 37].

The samples were small for any generalization but there was a tendency for readiness to be better in PNFP facilities where there were fewer clients compared to public health facilities. The supply chain of PNFPs is different from that of public facilities, and given that clients pay for services at these institutions, they may be able to demand for better services. Client satisfaction was also notably better in the PNFP hospitals. However, there are still challenges of quality of service provision in both settings. The human resource in both public and PNFPs facilities require attention if the quality of services is to improve. For instance, the lack of neonatal nurses for care of neonates deserves urgent attention. The newborn to midwife ratio of 1:20 in NSCU compared to the 1:4 in high income countries is appalling (7). Moreover these health providers are the same who deliver mothers in maternity units.

There were missed opportunities for immunization of the vulnerable babies before discharge. These could also be explained by the lack of vaccines, and/or lack of knowledge on when to vaccinate sick and small newborn babies [38, 39]. In addition, there were fewer mothers in PNFP facilities compared to public facilities who could mention at least two danger signs despite most of the mothers reporting to have been counseled, pointing to limited skills in counselling. Counselling of clients requires skilled professionals in that discipline. However, clinicians including nurses, though expected to undertake that role, are not experts in counselling and therefore may not consider it as a priority responsibility especially when they are overwhelmed by patient numbers. The HIV/AIDS service provision includes use of professional counsellors and this has notably improved the client’s awareness of the disease process and outcomes as well as adherence to medication [40]. Nonetheless, this is lacking in other areas of service provision like the maternal and newborn health. The MoH should pick lessons and consider recruitment of more staff particularly midwives, neonatal nurses, and counsellors in maternal and newborn health as it is currently doing for HIV/AIDs services.

The study findings showed that a significant proportion of babies were not assessed within one hour after birth. This may further suggest a high workload among health care providers. Every newborn baby requires thorough assessment in order to identify emergency complications that can be addressed, including failure to breath and hypothermia [11].

Notably, facility readiness for feeding was relatively good in all hospitals. Hospitals encouraged mothers to breastfeed, and used feeding cups or nasal gastric tubing when the babies were not able to suckle. The scores in this area could be explained by the recent concerted efforts of the PTBi project in the six hospitals through the clinical trainings and mentorships that prioritized monitoring and feeding of the sick and small babies in the NSCU. The project imparted knowledge and skills as well as provision of guidelines for each health facility. However, there were limited equipment and supplies provided at the beginning of the project [34] and these did not include items like breast milk pumps and infusion pumps. Feeding of vulnerable newborn babies may prove to be challenging due to the stressful situations mothers may be experiencing but also given the complications of the baby that may not allow suckling for some time. Notably, malnutrition is one of the risk factors for NDD in those babies [13]. Midwives require skills in this area in order to offer nutrients to the babies, and support the mothers in participating in effective and adequate nutrition of their babies.

Readiness for care of the preterm and LBW babies who are more likely to develop NDD is still lagging behind despite PTBi previous work in those facilities, and requires more attention. This implies that care for preterm babies elsewhere in district hospitals may be even worse, as evidenced by very low readiness scores for the HCIVs. There is considerable evidence that KMC improves not only survival of these neonates but also reduces risk of NDD [13, 41]. Moreover, practice of KMC is known to be challenging especially if the environment is not conducive [42]. Investment in KMC beds and chairs should be considered for district hospitals and HCIVs with designated KMC units. Furthermore, national guidelines for the NSCU in terms of lighting and sound control should be developed. There is evidence that highlights the relationship of the neonatal intensive care unit environment and NDD of vulnerable babies [43] and therefore needs to be addressed.

The results revealed that most cases admitted in newborn care units were due to asphyxia despite high facility readiness in resuscitation. This calls for urgent attention and investment in improving intrapartum care, particularly emergency obstetric and neonatal care. Evidence shows that availability of quality obstetric and neonatal care in countries with limited resources could prevent more than 50% of neonatal deaths and still births, and translate into better neurodevelopmental outcomes of the survivors [44, 45].

There are a number of policy implications of the findings from this study: The results reveal several readiness challenges in both hospital and HCIV facilities. The findings also showed that there are HRBs managed at HCIV level. There is therefore need for more investment in NSCU for comprehensive care of the sick and small newborn babies, beyond essential newborn care and resuscitation, in both hospital and HCIVs. This will improve quality service provision, reduce neonatal mortality and contribute to better neurodevelopment of infants. Resources are urgently needed, including human resource (numbers and skills mix: midwives, neonatal nurses as well as counsellors in maternal and newborn health), commodities and guidelines particularly for the care of preterm babies. In addition, health systems should be strengthened so as to provide quality intrapartum care/EmOC in order to reduce the numbers of asphyxia cases, and hence reduced burden of NDD.

### Study limitations

There are some limitations to the study: We were unable to do knowledge and skills assessment of health workers, yet availability of equipment and supplies may not translate into quality service provision without skilled human resource. We did not also assess for availability of vaccines given within the first week after birth which is a main factor for reducing early infections. In addition, few hospitals were included in the study limiting the generalizability of the study findings. Furthermore, exit interviews have an inherent weakness of social desirability bias, and mothers may have had recall limitations after a stressful child birth experience. However, the study findings are still important in informing policy and programming for improved service delivery aimed at transforming care for improved neurodevelopment of the most vulnerable newborn babies.

## Conclusion

The results revealed low health facility readiness scores in both hospitals and higher level health centres (HCIVs), particularly for the care of preterm babies. In addition, the main cause of admission to the NSCU was birth asphyxia. There is therefore need for more investment for comprehensive emergency obstetric care and care of the sick and small newborn babies in both hospital and HCIVs. This will contribute to improvement in the quality of maternal and neonatal care, reduce neonatal mortality, and result in better neurodevelopment of infants.

## Data Availability

The datasets generated and analyzed during the current study are not publicly available due to the fact that some of the data were obtained from government of Uganda health facilities, but are available from the corresponding author on reasonable request.

## Abbreviations

AIDS: Acquired immune-deficiency syndrome
ANC: Antenatal care
CPAP: Continuous positive airway pressure
ECD: Early child development
EmoC: Emergency obstetric care
EmoNC: Emergency obstetric and neonatal care
HBB: Helping babies breathe
HCIVs: Health centre IVs
HDREC: Higher degrees, research and ethical council
HIV: Human immunodeficiency virus
HRB: High risk newborn babies
KMC: Kangaroo mother care
LBW: Low birth weight
LICs: Low income countries
LMICs: Low and middle income countries
MoH: Ministry of Health
NDD: Neurodevelopmental disability
NSCU: Neonatal special care unit
PNFP: Private Not for Profit
PTBi: Preterm Birth Initiative
QoC: Quality of care
RRH: Regional Referral Hospital
SARA: Service availability and readiness assessment
WHO: World Health Organization
